# Information Flow between Parents and Offspring Is Essential for Successful Development

**DOI:** 10.3390/biom13091299

**Published:** 2023-08-24

**Authors:** William V. Holt, Joaquin Gadea

**Affiliations:** 1 Department of Oncology & Metabolism, The Medical School Beech Hill Road, Sheffield S10 2RX, UK; 2 Department of Physiology, International Excellence Campus for Higher Education and Research “Campus Mare Nostrum”, University of Murcia, 30100 Murcia, Spain; jgadea@um.es

Over the last several decades, the sciences of developmental biology and physiology have expanded and intertwined their scope enormously. Where once it might have been acceptable to believe that a zygote (derived from a single egg and a spermatozoon) is just an undifferentiated but autonomous cell that has charge of its own future, this is no longer the case. It is increasingly evident that the future health and wellbeing of developing embryos, regardless of species, depends on the health, nutritional status and even psychological status of the previous generation(s). How is it possible that the future growth, phenotype and survival of such a zygote could be entirely encoded in its genes? Developmental biologists have struggled with this question for millennia, and over the years, various hypotheses have been proposed and later rejected because they could not provide a satisfactory explanation (for review, see [1]). One of the more recent approaches to this topic, the reductionist approach, gained popularity once researchers appreciated the functional significance of genes as both components and controllers of biochemical pathways. This rather inflexible and mechanistic paradigm of development was itself challenged by the same author [1], who argued that the reductionist approach is too focused on identifying molecular pathways and signal networks and fails to appreciate the processes which lead to phenotypic diversity, complex traits and their variations.

These different opinions reflect the divergent backgrounds and prejudices of the investigators themselves, and the small collection of articles in this Special Issue demonstrates clearly that an understanding of development involves inputs from a multitude of different sources and disciplines. Here, we have taken a broad approach, which not only demonstrates the developmental importance to adult health (in mammals) of being provided with good quality maternal and paternal diets before conception [2], but also how stress in fishes, via parental ethanol exposure prior to conception, can dramatically affect the behavior of offspring. The rationale for this particular study [3] was to see whether the mechanisms behind “Foetal Alcohol Spectrum Disorders (FASD)” in humans could be investigated using zebrafish development as a model system. Stress responses in larvae produced by adult females that had been exposed to 1% ethanol, as well as an alarm cue (an extract prepared from the skin of stressed fishes), were less sensitive to the alarm cue than those derived from control females not exposed to ethanol. It is apparent from this study that environmental conditions, such as both ethanol exposure and exposure to the stress cue, experienced by adult females have lasting consequences on the subsequent generation. Presumably, the underlying cause of what seems to be a psychological effect is being produced via perturbations of brain function.

Additionally, in this Special Issue, Davies et al. [4] investigated a different role of stress hormones (glucocorticoids), where, in late pregnancy, they help to satisfy the increased energy needs of the mammalian brain as it adapts to new postnatal functions. In essence, the brain adapts by generating more mitochondria and producing more ATP via oxidative phosphorylation. However, this description is an extreme simplification as the brain is such a complex organ, and the authors showed that, in reality, the different anatomical regions of the brain do not all respond in the same way. 

Two of the papers published in this Special Issue [5,6] explore communication mechanisms between cells and tissues, including embryo-maternal interactions within that sphere, focusing primarily on the importance of extracellular vesicles (EVs). Over the last few decades, it has become increasingly obvious that cells communicate with each other by packaging important molecules within EVs of various dimensions and releasing them into situations where they can be recognized and internalized by their target cells. One of the first such recognized interactions involved the transfer of EVs from epididymal cells to spermatozoa undergoing maturation within the epididymis [7]. Since then, EVs have been analyzed in detail using mass spectrometry and are now known to contain many proteins and diverse species of RNA [8]. The two EV studies reported in this Special Issue confirmed that EVs function to modulate the physiology of the mammalian female reproductive tract, and besides participating in embryo development and implantation, they can be used to enhance in vitro embryo development and modulate embryo transcription after embryo transfer [5,6]. Both articles present data that relate closely to the review about the debilitating impacts of endometriosis in women [9], a major cause of human infertility. Endometriosis is a pathological condition involving many of the same processes as implantation, and the authors discuss the significance of genetics, epigenetics, glycosylation and microRNAs.

The nutritional relationships between parents and offspring in teleost fishes have, in the past, received a great deal of attention, mainly in the context of food production and growth rates [10,11], but also in more specialized fields, such as seahorse and pipefish conservation [12,13,14]. However, as discussed by one of the articles in this Special Issue [15], because fish development can be supported entirely from the maternally supplied yolk (lecithotrophy) or supplemented after fertilization from maternal (matrotrophy) or paternal (patrotrophy) sources, there are some inconsistencies in the way that embryo nutrition is defined and measured across different studies. The authors recommend that the use of an alternative and more objective approach to obtaining a parentotrophy index is essential to reliably distinguish between provisioning strategies in teleosts.

Finally, the paper by Holt and Comizzoli [16] takes a broad view of reproductive biology, focusing on recent progress in epigenetics and asks whether the rapidly changing environment might bring about developmental changes akin to accelerated evolution. The field of developmental epigenetics is still in its infancy, but it could resolve some very subtle but important and transgenerational effects. Epigenetic transgenerational relationships are now being implicated in the field of nutrition, where grandparental nutrition is known to influence the growth and fitness of grandsons and granddaughters (see Batra et al. in this Special Issue [2]). However, the susceptibility of small rodents to flea infestations [17] has recently also been shown to depend on the extent of grandparental flea exposure. It is likely that explaining such effects as parent–offspring effects still requires more inputs from diverse scientific disciples, such as olfaction, pheromone detection, animal behavior and perhaps all the way through to sexual selection and evolution. The main lesson from this small collection of manuscripts is, therefore, that researchers are not running out of topics to investigate!
